# The social ecological model of interpersonal health communication among rural older residents: a consensual qualitative study

**DOI:** 10.3389/fpubh.2025.1615088

**Published:** 2025-08-05

**Authors:** Weijuan Kong, Cailing Yang, Xinjin Li, Yanhua Ning, Jing Shi, Lingna Liu, Haiyan Liu, Yahong Guo, Meiman Li

**Affiliations:** ^1^Department of Master’s Training Station, General Hospital of Ningxia Medical University, Yinchuan, China; ^2^School of Nursing, Ningxia Medical University, Yinchuan, China; ^3^Department of Nursing, Wuzhong People’s Hospital, Wuzhong, China

**Keywords:** older people, rural area, interpersonal health communication, family doctors, consensual qualitative research

## Abstract

**Objectives:**

To explore the ecology of interpersonal health communication behavior among older residents in rural areas of western China.

**Methods:**

Adopted semi-structured interviews with 15 older residents and 15 family doctors in rural areas of five prefecture level cities in Ningxia Hui Autonomous Region, China. Used the Consensual Qualitative Research method for data analysis. Based on the ecological map drawn, understand how the interpersonal health communication behavior of older residents is carried and what factors affect it in rural area.

**Results:**

According to the Ecological Systems Theory, we identified following themes: Microsystem (Weak recognition of health knowledge needs, Informal communication: mutual communication between relatives and friends and formal communication: one-way communication of family doctors); Mesosystem (Multi scenario and Multi channels); Exosystem (Medical environment and Family doctors’ workload) Macrosystem (National policies, Regional characteristics and Living environment).

**Conclusion:**

In summary, the health communication mode of older residents in rural areas is different from other populations, with face-to-face communication based on family doctors being the dominant mode and influenced by multiple levels of factors. Therefore, it is necessary to establish systematic plans at the policy, organizational, and other levels, especially in the training of family doctors, so that they can carry out more extensive and deeper professional interpersonal health communication, enhance the health literacy of the older, and maintain their health level.

## 1 Introduction

Health communication is any type of human communication whose content is concerned with health and it can take many forms (1). Interpersonal health communication is mainly reflected in the communication and interaction between patients, doctors, and nurses when seeking medical treatment (2). The advantage of interpersonal health communication is that organizations providing medical services, medical personnel (doctors and nurses), and auxiliaries (nurses, administrative staff, security guards) add value in healthcare through the way they interact with people (3). Interactions around health topics are not isolated events but rather occur in various patterns of social interactions that are longitudinal and iterative. Meaning making around health topics is constructed, shared, elaborated, reconstructed, and interpreted in participants’ social networks, as information is distributed through a complex temporal system of interpersonal ties (4). In rural settings, local networks have a strong influence on the spread of health information (5), the current mode of health communication among rural residents mainly relies on publicity and education (6). In current China, rural mainly refer to settlements of people engaged in agriculture, forestry, animal husbandry, fishing and other industries (7). Family doctors bear the important responsibility of disseminating health information to rural residents. They provide primary care services to the rural population under the management of township health centers (8, 9), and increase service accessibility and access to high-quality healthcare, particularly for individuals living in rural areas (9).

With the acceleration of global aging, coupled with a significant gap in health between older residents in rural and urban areas (10), rural older populations face compounded health challenges due to medical resource disparities and mobility limitations. Actively seeking health services lies at the core of effective models of chronic disease self-management and contributes to promoting the utilization of health services (11). The acquisition of health knowledge is crucial for improving health literacy among the older. For older residents in rural areas, exchanging health information through interpersonal communication is an effective healthcare activity to improve their health status (12). Internet technology have been widely used, however, according to the data of the 2017 China Comprehensive Social Survey, due to the supply barrier of Internet equipment and insufficient resources, rural older people are limited in using the Internet to obtain health information (13), they still obtain health information through interpersonal communication. Family doctors, often the primary healthcare providers in these areas, play a critical role in shaping the health outcomes of older residents through interpersonal health communication. Their interpersonal health communication behaviors directly shape older residents’ health literacy, treatment adherence, and preventive care practices.

Based on the data from the 2020 China Family Tracking Survey, Chinese scholars have found that interpersonal relationships, WeChat usage, and regional distribution are influencing factors of older people’s self-rated health (14). Additionally, factors such as age, monthly income, living area and self-reported good health were more likely to influence health in middle-aged population (15). However, most existing studies (including above findings) have mainly focused on single-level analyses, and using social ecology model to explore the influencing factors of interpersonal health communication behavior among older people in rural can systematically analyze from multiple levels and dimensions.

**Figure 1:**
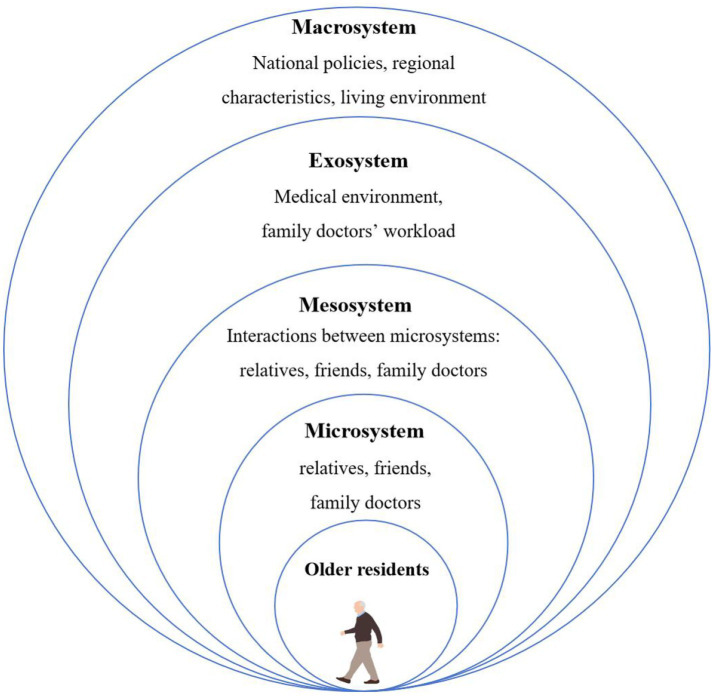
The ecology of interpersonal health communication behavior among older residents in rural area. **Drawn by referencing relevant literature (17, 42).

Ecological Systems Theory (EST) is Bronfenbrenner’s observation of children’s behavior and their interactions with familiar adults in the natural environment, it consists of five layers, and within each layer are developmental processes unique to the layer, and relationships and interactions prototypical to the layers along with key factors unique to a layer (16, 17): (a) microsystem: family and siblings and immediate physical home environment; (b) mesosystem: the neighborhood, school, church, and parks; (c) exosystem: parent–guardian workplace, fire department, welfare system, police, health care, and other forms of family social support; and (d) macrosystem: the outermost layer or incorporates the local, state, and national government narratives, ideologies, and social policies. According to EST, human behavior can be considered in terms of a hierarchy of related systems with interactional patterns between and within the systems. Therefore, this study divides the interpersonal health communication behavior of rural older residents and their interaction with surrounding people and environment into five levels based on this theory, and explores the typical relationships and interactions related to different levels (Figure 1).

As we can see, older residents play a core role in interpersonal health communication, while relatives, friends, and family doctors are located in the microsystem. The mesosystem refers to the interaction between the microsystem (including friends and rural doctors) and older people. The exosystem can be seen as an extension of the mesosystem and includes both formal and informal social structures that influence and delimit the person (18), it mainly refers indirect influence like medical environment and workload of family doctors in this study. The macrosystem refers to the wider societal and cultural norms, including national policies, regional characteristics and living environment.

Effective communication between family doctors and older residents is essential for promoting health literacy, ensuring treatment adherence, and facilitating preventive care (19). However, the dynamics of these interactions remain under-explored, particularly in rural settings where sociocultural and structural factors significantly influence communication patterns. Due to the characteristics of acquaintance society in rural areas, individuals exhibit strong mutual dependence and frequent interaction, family doctors are members of an acquaintance society (20). Older people turn to their personal networks when seeking information and support, and they prefer ‘word of mouth’ communication, interpersonal interaction between older individuals and acquaintance is beneficial for health communication (21). However, there is a knowledge gap in the dynamic interpersonal relationships between family doctors and older people. Understanding this dynamic interpersonal interaction can help older people express their health needs more accurately and assist family doctors in providing targeted interpersonal health guidance.

Consensual Qualitative Research (CQR) is a descriptive, inductive research method based on data collected through interviews involving open-ended questions and a semi-structured format (18, 22). Exploring naturally occurring phenomena and collecting data through interactive interviews with participants, deriving the meaning of the phenomenon of interest from their discourse and text, paying attention to the language context of the participants, exploring and clarifying the data, and having 3–4 team members review the data from multiple perspectives can ensure in-depth exploration of the research phenomenon (23). Interpersonal health communication is carried out through interaction between individuals, therefore the personal feelings of communicators and recipients are the most direct evidence reflecting the transmission situation. For a more nuanced understanding of older residents’ and family doctors’ subjective experiences, it is important to complement the quantitative studies with open-ended qualitative queries. Specifically, based on the concept of EST, using the CQR methodology, we aimed to conduct in-depth interviews to explore the reality of interpersonal health communication behavior among older people in the microsystem, understand how older rural residents receive, communicate, and utilize health information during interpersonal interactions with doctors, family members and friends in the mesosystem, and analyze the impact of medical resources and macro policies on the interpersonal health communication behavior of older people in the exosystem and macrosystem.

## 2 Methods

### 2.1 Research design

This study used a CQR design exploring interview data. This design was chosen as it allowed us to examine how understanding the experience and feeling of interpersonal health communication between older people and doctors in rural areas might be socially constructed in a natural setting. The reporting of results adhered to the COREQ guidelines.

### 2.2 Participants and procedure

#### 2.2.1 Participants recruitment

**Figure 2:**
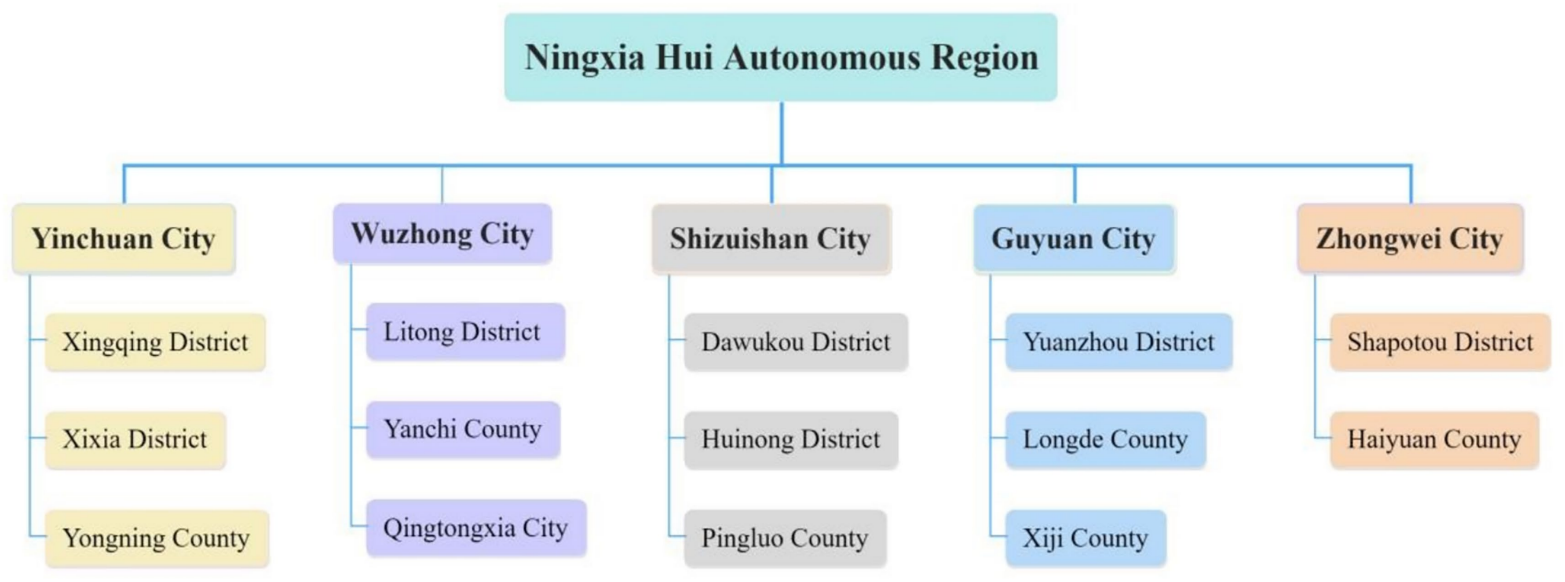
Research objects selection source.

Given that family doctors are the most professional communicators of health knowledge in rural areas, and older residents are the main audience and secondary communicators. This study recruited two key informant groups: older residents and family doctors in rural areas of five prefecture level cities in Ningxia Hui Autonomous Region. From February to November 2023, semi-structured interviews were conducted with the aforementioned groups. The main sources of participants are shown in Figure 2.

The inclusion and exclusion criteria for the research subjects were as follows: Older residents’ inclusion criterion was that they had lived in rural areas for at least 1 year and aged 60 years or above. The exclusion criteria included those with speech or hearing impairments, communication difficulties, severe liver or kidney dysfunction, cancer, or end-stage diseases, or poor mental state. Family doctors’ inclusion criterion was that they possessed the rural doctor qualification certificate and had engaged in rural health work for at least 1 year. Those doctors who went out for learning or further education, engaged in hospital management, were lost to follow-up by the researchers, unable to provide feedback, or requested to withdraw from the study were excluded. All participants voluntarily participated in this study after knowing the research purpose.

Based on the sample size requirements for CQR (18, 22), this study selected 15 older rural residents and 15 family doctors for data collection. A total of 35 interviews with 30 participants voluntarily participated in this study. We used the purposive sampling strategy of maximum variation to enroll participants residing in rural areas of Ningxia. Older people were diverse in terms of gender, age, education level, income and marital status while doctors were diverse in terms of education level, professional title and working hours (see Table 1).

**Table 1:** Demographic profile.

Older residents	N(%)	Family doctors	N(%)
Gender		Gender	
Male	8(53.3)	Male	6(40)
Female	7(46.7)	Female	9(60)
Mean age (years)	M = 69.3 (min = 60; max = 84)	Mean age (years)	M = 43.9 (min = 33; max = 58)
Education		Education	
Uneducated	4(26.7)	Secondary specialized school	4(26.7)
Elementary	7(46.6)	Junior college	9(60)
Intermediate and above	4(26.7)	Bachelor’s degree or above	2(13.3)
Occupation		Professional title	
Physical labor	12(80)	No	3(20.2)
Mental labor	3(20)	Assistant rural physician	6(40)
		Attending rural physician	6(40)
Marital status		Work hours (years)	
In marriage	10(66.7)	<10	2(13.3)
Widow/Divorce	5(33.3)	10~20	4(26.7)
		>20	9(60)
Monthly income(CNY)		Works hours for health communication	
<1,000	5(46.4)	<10	3(20.2)
1,000–3,000	8(53.3)	10~20	9(60.6)
>3,000	2(13.3)	>20	3(20.2)
Number of Children			
0	2(13.3)		
1~3	10(66.7)		
>3	3(20.2)		

#### 2.2.2 Interviewers and research team

Interviewers were three nursing graduate students and a experienced community nursing research professor from research team (five nursing graduate students, a grassroots general practitioner, and two experienced community nursing research professors). They practiced the interview guide and conducted recorded mock interviews; feedback was provided on above process to ensure consistency across interviewers. Participants provided informed consent before the interview. The interview recordings were deidentified and transcribed by three interviewers.

#### 2.2.3 Data collection

Interview guide

Based on the research purpose and content of this study, and with reference to the Comprehensive Model of Information Seeking (CMIS) and the Interpersonal Needs Theory (INT), two graduate students engaged in research related to health communication in rural areas initially developed the interview guide. After internal discussions within the research team, communication and nursing experts were invited to modify the guide. In December 2022 and January 2023, two older rural residents and family doctors were selected for pre interviews to verify the feasibility of the research plan and interview guide. After that, researchers organized and analyzed the interview data, improved the interview guide, and final interview guide is shown in Table 2.

**Table 2:** Participants interview guide.

Older residents	
No.	Interview question
1	How is your current health condition? Will you actively pay attention to health/wellness related information?
2	Do other people usually communicate health or wellness information to you? Could you please provide more details? (such as family doctors, community nurses, family and friends, etc.) (including the methods, main content, time, experience)
3	How effective do you think they are in communicating health or wellness information to you? Specific manifestations?
4	Do you communicate health or wellness information to your relatives, friends, or family members? Can you please provide specific details about your experience?
5	How do you experience communicating health or wellness information to others? Can you explain in detail?
6	Do you have anything else you would like to add?
Family doctors	
No.	Interview question
1	How do you usually carry out health information communication to older residents? Could you explain in details?
2	How did you experience communicating health information to older residents?
3	What difficulties do you think exist in communicating health information (face-to-face/through media) to older residents in rural areas? What is the specific reason?
4	What factors do you think will affect your communication of health information to older residents?
Supplementary questions	
No.	Interview question
1	How to promote active participation of older people in health communication?
2	What are the differences in the health promotion for the older compared to other groups of people?
3	What can be further developed in the communication of health information to the older by village clinics, and what conditions need to be provided?
4	Do you have expectations for the communication of the geriatric health knowledge that you are responsible for, and do you have any relevant suggestions?
5	Do you have anything else you would like to add?

The interview guide was not fixed, and adjustments would be made during the interview process based on the answers provided by the interviewees. Before each interview, we contacted the interviewee first, informed them of the main research content, and determined the interview location and time in advance. We prepared an informed consent form, an interview script, and a general situation survey form for the interview. The entire interview process was recorded using a mobile phone. We carefully observed and recorded the interviewees’ emotional and behavioral responses.

#### 2.2.4 Data analysis

We followed the qualitative framework of Consensual Qualitative Research (CQR) to analysis the interview data. The process of developing codes using this method is as follows: developing and coding domains, constructing core ideas, and forming categories to describe consistencies across cases (23). According to CQR guidelines, research team members should reach a consensus regarding all analytical decisions (24).

In the first step, members of the research team developed a list of domains based on the interview guide and literature. They then independently coded the transcripts and reviewed the interview transcripts coded by the others until the research team reached a consensus on the domain list through discussion.

In the next step, during construction core ideas, team member identified subthemes in different domains by repeatedly reading interview transcripts. The domains and subthemes were coded by multiple members. After all the transcripts were coded, team members read all the excerpts under each domain, summarized them into core ideas, and reached a consensus after judges reviewed these core ideas.

In the final step, three judges reviewed the results to ensure that the raw data were categorized accurately and that the core ideas appropriately reflected the interview data.

#### 2.2.5 Quality control

Prior to the interview, the researcher contacted the interviewee and informed them of the purpose of the interview. The interview location will be chosen by the interviewee themselves, considering quietness and privacy. During the interview, we carefully listened to the interviewee’s expression, encourage them to express their true thoughts, and recorded the content they expressed, body movements, facial expressions, etc. After transcribing the interview data, confirmed with the research subjects again whether their expressive content is fully recorded. The research team uses consensus to construct their interpretation of the data, trying to set aside their biases so that they fairly describe what the participant has reported.

## 3 Results

### 3.1 Microsystem

Microsystem including relatives, friends, and family doctors. The most important is to firstly clarify the current situation of interpersonal health communication behavior among older residents in rural areas, and then explore the professional health communication behavior of family doctors and their impact on the interpersonal health communication behavior of the older residents.

#### 3.1.1 Weak recognition of health knowledge needs

According to the ecology of interpersonal health communication behavior among older rural residents drawn from relevant literature, older people are at the core of communication behavior. Therefore, older adults’ personal awareness is a key factor influencing communication behavior. In recent years, the health awareness of older rural residents has gradually evolved, but this study found that some older people in rural areas still exhibit weak health awareness, insufficient recognition of health knowledge needs, and a lack of enthusiasm for participating in health communication.

*O3: “Mainly because I’m getting older now, I feel like paying attention to health communication doesn’t have much effect anymore.”*

*O4: “We are getting older, and paying attention to health now actually doesn’t have much effect.”*

The above statements all reflect that older people, as a core part of the ecology of interpersonal health communication behavior among older residents, has a weak recognition of health knowledge needs. This precisely reflects the research purpose of the reality of interpersonal health communication of older residents.

#### 3.1.2 Informal communication: mutual communication between relatives and friends

In the microsystem, relatives and friends as the group with the closest relationship with the older people, often engage in mutual communication behavior in daily life, but this behavior is often unconscious or based on their own experience and hearsay, and communicate to each other during conversations. This can be referred to as informal communication, forming an important part of interpersonal health communication.

Most older residents mentioned that the communication of health information from relatives and friends is only done through casual conversations at home or through remote communication devices. The communication content mainly focuses on daily life related to clothing, food, housing, and transportation, occasionally involving health-related knowledge related to diseases.

*O8: “My family members also tells me about it (health communication), reminding me to pay attention to eating and drinking in daily life, not to eat raw and cold food, to wear more clothes when it’s cold, and to be careful of catching a cold.”*

*O15:* “*When I often gather with friends to chat, we talk about who got sick, how they got sick, and so on.*”

Informal communication, as a microsystem in the social ecology, mainly involves the dissemination of daily life essentials such as clothing, food, housing, and transportation. Although informal communication is not highly professional, this indicates that it is also an indispensable component of the current interpersonal communication landscape among older adults.

#### 3.1.3 Formal communication: one-way communication of family doctors

Similarly, there is another important group in the microsystem, family doctors. As professional health personnel, the communication behavior of family doctors can be referred to as formal communication in interpersonal health communication.

The process of health communication is to convey health-related information such as knowledge and mental health to the public. As an important component of health communication, family doctors carry out interpersonal health information communication with older residents in rural areas first out of their professional requirements, focusing on disease prevention and daily life care for the older.

*Y6: “Tell older residents about the daily precautions for common chronic diseases such as hypertension and diabetes, which mainly involve diet and medication.”*

*Y9: “When older people come to the clinic for medical help, I also communicate some health-related information to them based on my professional knowledge.”*

*O7: “The family doctor taught us how to have a reasonable diet, lose weight, and so on.”*

*O15: “The doctor in the village, when we went for a physical examination, he told us to eat light oil, light salt, and eat more vegetables.”*

Health communication from rural doctors is an important source of scientific knowledge for older adults. Statements from both rural doctors and older adults indicate that health communication in the microsystem by rural doctors mainly focuses on disease prevention and the healthy lifestyles advocated by the government.

### 3.2 Mesosystem

#### 3.2.1 Multi scenario

The mesoystem mainly involves the interaction between relatives, friends, family doctors, and older people in the microsystem, presenting various scenarios and patterns.

Informal health communication between family and friends mainly occurs during daily conversations.

*O4: “My daughter and grandson often explain these things to me, and we talk about them when we chat at home.”*

*O8: “When people in the village gather together to chat, they will talk about health information spread by family doctors, including disease prevention and other related topics.”*

The most critical interactive factor in this system is the interpersonal health communication between village family doctors and older people. Which is carried out in various contexts, mainly includes specific Health Days, physical examinations, home follow-up, and when older residents come to the clinic for medical treatment.

Specific Health Day is a health communication activity organized by family doctors around a specific theme for older residents. Such as the Hypertension Day, Stroke Day, etc.

*Y1: “The health communication combined with some large-scale festivals, such as the Tuberculosis Day*, *we are all doing corresponding health education.”*

*Y3: “We can use various public health services to carry out corresponding health knowledge publicity on special days such as the Hypertension Day and diabetes Day.”*

China’s national basic public health service project stipulates that free physical examinations, including health management services, must be provided to older people every year. Family doctors happen to use health examinations to disseminate corresponding health information to the older, and will also provide timely feedback on the examination results after the examination is completed.

*Y11: “During the annual free physical examination, we spread some health knowledge to the older residents.”*

*Y13: “We conduct health checkups for older people every year. After the results of the checkups are available, we also provide feedback to them, and we also give corresponding education.”*

*O12: “After physical examinations, I went to the clinic to measure my blood pressure. The family doctor said that it seemed that there was gallbladder cyst and advised me to avoid eating fatty meat.”*

Home follow-up is also an important part of the daily work of family doctors, and health communication behavior runs through it.

*O9: “Like the doctor in our village (family doctor), he will come to my home whenever he has time, sign a book (Family Doctor Signing Book) for us, and tell us how to prevent high blood pressure.”*

*Y4: “I will pay a visit to older people’ home at least once a quarter. During this period, I also give corresponding guidance to each older according to their different physical conditions.*

The village clinic is the closest medical service institution to rural residents, and most rural patients choose to seek medical treatment here. Whether it is buying cold medicine or commonly used drugs for chronic diseases, or when need diagnosis and treatment for daily physical discomfort, older residents will come to the clinic to seek the help of family doctors. During the process of seeking medical treatment, family doctors not only provide disease examination and medication services for older residents, but also promote corresponding health information to them.

*Y7: “When older people come to the clinic for medical treatment or to buy medicine, there will be an outpatient follow-up to guide them on how to exercise, eat healthily, and take medication, and so on.”*

Besides the clinic, visiting other hospitals is also one of the channels for older residents in rural areas to obtain health information.

*O5: “When I went to hospital outside to get medicine, doctors told me not to eat too salty, to eat more vegetables and fruits, and to get enough sleep and exercise more.”*

From the statements of the two groups of respondents, it can be seen that the informal interpersonal health scenarios in this system are relatively single, while formal interpersonal health communication occurs in various scenarios.

#### 3.2.2 Multi channels

Both formal and informal communication involve interpersonal health communication with older residents through multi channels, including remote communication with capable older people using electronic tools such as WeChat, and through the multidisciplinary team of good cooperative relationships within teams by family doctors.

In the context of rapid development of mass media, family doctors can use mass media to communicate health information more conveniently and quickly. However, some older people have limited ability to participate in interpersonal health communication through the media, making it difficult for them to receive such communication.

*Y11: “Some older people don’t have WeChat and only use those old phones. They can only accept face-to-face explanations to spread the message when they come for treatment.”*

*O6: “Usually I can answer it if someone calls me, but when they send me a WeChat videos or articles, I don’t watch it because my eyes won’t be able to see clearly.”*

*Y8: “Each member of the family doctor team has their own division of labor. With the help of the higher-level doctors, we each are responsible for different health communication content*.*”*

*Y6: “When I saw that the WeChat official account, or those health knowledge related to the older publicized by the township health center, will basically be forwarded to the livelihood service group in our village.”*

The participants’ statements clearly demonstrate that, to a certain extent, insufficient capabilities of mass media can have a negative impact on health communication. And with the help of multidisciplinary team collaboration, it is more helpful for family doctors to carry out interpersonal health communication.

### 3.3 Exosystem

#### 3.3.1 Medical environment

The medical and health facilities at the grassroots level are inadequate, this makes it difficult for residents to obtain more drugs and to diagnose diseases, which reduces the frequency of older people going to the clinic for medical treatment. Consequently, the communication related to health knowledge between doctors and older people is reduced.

*O2: “The medical resources in the village are also a bit lacking. Many times, the village clinic or even the health center cannot meet the needs of the patients, and they have to seek medical treatment from hospitals in the county and city.”*

*O11: “I also rarely go to those health-related activities in the village clinic. For physical examinations, my son who takes us both to the city for the check-up, and we don’t go to the village check-up.”* if this is because primary health care is often not ideal for treating complex, chronic diseases and requires a multidisciplinary team.

As one of the important places for rural residents to seek medical treatment, village clinic is an important component of the older medical environment. The distance from the clinic also affect the willingness and frequency of older residents to seek medical treatment, which in turn affects the frequency of receiving health information dissemination.

*O13: “We’re not far from the village clinic. Sometimes when we come across that doctor, he also tells us about health related information.”*

*O14: “The village clinic is quite far away. If we go there, we also arrive at the township health center. So why don’t you go to the center instead of seeing the family doctor?”*

The choice of medical location by rural older residents directly affects their interpersonal interaction with family doctors and other medical personnel. The medical environment is a factor influencing the interpersonal communication behavior of older people in the exosystem.

#### 3.3.2 Family doctors’ workload

Family doctors not only provide public health services and basic medical services to rural residents, but also undertake other related tasks entrusted by the health administrative department, including health records management and support for township health centers.

*Y2: “I am the only one doctors in our village clinic, and sometimes I need to go to the township clinic for support.”*

*Y12: “The workload actually is the largest. For example, in this village, there are currently thousands of people, including hundreds of older people. This workload is actually very, very large (in health communication).”*

*Y3: “Family doctors are very tired, because we are also engaged in outpatient services. Besides, I am in charge of more than 120 hypertension (patients), more than 30 diabetes (patients), and nearly 200 older people. Some of them can’t really get services.”*

In this system, family doctors have extensive work responsibilities and high work pressure, which leads to a corresponding reduction in the time spent on health communication and affects the frequency of information transmission.

### 3.4 Macrosystem

The main influencing factors of interpersonal health communication for older residents from the macrosystem including national policies, regional characteristics, living environment, medical environment.

#### 3.4.1 National policies

With the continuous deepening of aging, multiple policies have been introduced at the national level to promote healthy aging. According the statements of older residents and family doctors, which provides convenience for family doctors to communicate health information to older residents at a macro level.

*Y6: “Now the platform provided by the government has also been given, and the investment is relatively large. The medical policies are also good, and those who have medical insurance can be reimbursed for medical treatment. People are more willing to come to see a doctor.”*

The public health service project also requires family doctors to carry out health communication for residents. Most family doctors mentioned that in the process of carrying out health communication for older residents, the signing of family doctor services in the local area is relatively good, and they can actively use this form to carry out related work.

*Y1: “There is a family doctor contract service, which means we go there once or twice a year to sign this contract. After that, if residents have any problems, we can also solve them accordingly.”*

#### 3.4.2 Regional characteristics

People living in the same area may have common collective behavior, and as the most direct embodiment of regional characteristics, individual behavior will be influenced by regional characteristics. Older residents may be the group with the longest residence time in a certain area, and whether as passive recipients or active communicators in interpersonal health communication, they will be more or less influenced by regional characteristics.

*O7: “No one in our village pays attention to health knowledge, and the whole village basically doesn’t care about it, so I neither.”*

*Y1: “It’s related to the overall atmosphere of each community. The older people in my jurisdiction have a high overall enthusiasm for participating in health education.”*

The subtle influence of regional characteristics exists in the behavior of older people participating in interpersonal health communication. When people around actively discuss health knowledge, most older people are attracted to participate. However, such indifference toward health knowledge can negatively impact their participation in health communication.

#### 3.4.3 Living environment

Whether older people live with family members and whether the living environment is convenient for older residents to participate in social and interactive activities, which is the important foundation for the occurrence of interpersonal health communication behavior.

*O10: “I live in front of the river channel. There’s hardly anyone there, so I came over here to chat with other people.”*

*O1: “Yes, it’s all face-to-face to talk(health related information), because we also live together, so we often talk about it.”*

It indicates when the living environment of the older people is conducive to communication with the outside world, the transmission frequency will increase, while conversely, the transmission frequency will decrease.

## 4 Discussion

This study discussed the reality and influencing factors of interpersonal health communication behavior among rural older residents, based on an ecology model of interpersonal health communication behavior among rural older adults. It is clear that older residents occupy the most central position in communication. From the perspectives of microsystem, mesosystem, exosystem, and macrosystem, we explored the reality of interpersonal health communication among rural older people, the role of family doctors, and their impacts on interpersonal health communication behavior.

In microsystems, we have clarified the current status of interpersonal health communication among rural older adults. Older residents show weak recognition of health knowledge needs, primarily because factors like cultural level and economic status lead to low proactive health consciousness and a lack of health concepts (25, 26). Informal communication from family and friends is a key component of such communication, featuring mutual interaction—this aligns with rural China’s reality, where health information spreads mainly through face-to-face interactions, such as daily greetings, casual conversations, or sharing medical-seeking experiences (27). Besides, family-provided emotional support and daily care in intergenerational support have positive effects (28). Thus, health education for older adults and their families should be strengthened (29). This study finds that formal communication from family doctors is one-way: older adults receive information passively without active feedback, though they reflect on the content and decide whether to adopt it based on their needs. As the closest medical providers to rural residents (30), family doctors play vital role in health information dissemination, focusing on disease prevention and daily care for older adults. Consistent with previous research, village doctors provide basic healthcare services for the elder, and take care of issues such as colds, diarrhea, and stomachaches, and they also provide public health care, health prevention, and rehabilitation (31).

In Mesosystem, we explained how the interaction between the microsystem (including relatives, friends, family and doctors). The interpersonal communication behavior between relatives, friends and older people mainly occurs during daily conversations, because there are often close family or neighborhood relationships between people in rural communities. Family doctors carry out interpersonal health communication for older people in multi scenarios including specific health day, free physical examinations, home follow-up visits, and seeking medical advice (32, 33). Conducting free consultations and health education for corresponding diseases on Health Day has become a means of health communication, spreading different health information on different Health Days. In China, annual physical examinations are provided free of charge for adults aged 65 and above (34). Family doctors disseminate basic health information on site during physical examinations, and after the examination is completed, village doctors can disseminate corresponding health information based on their own health status when providing feedback on the examination results. Home follow-up as one of the working forms of village doctors, home follow-up also provides strong support for the health communication of the elder. Older people often go to village clinic for visited by who report experiencing headaches, colds, or purchasing medication to seek medical help from family doctors. Doctors will also use this time to promote health information to them.

Besides, relatives and friends, as well as family doctors, engage in interpersonal health communication with the older people through multi channels such as mass media and family doctor signing service. Relatives, friends, and family doctors use information tools such as WeChat to communicate health information online with some older people. With the rapid development of mass media, older people prefer to use WeChat to obtain and share daily health information (35). Previous study pointed out that internet use has a significant positive impact on the health of middle-aged and older adults (36). In 2016, the Chinese government fully implemented the family doctor signing system (37), prioritize the coverage of older people and other. The team usually consists of general practitioners, nurses, and public health doctors, becoming an important model for family doctors to communicate health information to older residents. Rural China is hugely dependent on traditional healers rather than groups of doctors, especially for older people. Family doctors usually establish long-term trust relationships with rural older residents, and the two parties become more familiar with each other (27). This familiarity allows older people to place more trust in family doctors during diagnosis and treatment. In contrast, medical teams composed of doctors from other hospitals or regions lack familiarity with local seniors, which may limit the older people’s access to diverse medical resources. Therefore, it is necessary to further promote family doctor services in rural areas.

In exosystem, the health environment and the workload of family doctors have an impact on interpersonal health communication. The impact of medical environment on the health communication behavior of older residents is mainly reflected in the choice of medical facilities and the distance between village clinics. The outdated infrastructure of village clinics and incomplete drug allocation affect residents’ frequency to seek health advice from family doctors. The distance to the health clinic will also have the same impact. This is because the travel distance of residents in small and medium-sized cities in China is the primary consideration for daily medical care (38). In addition to a large amount of routine workload, village doctors also need to complete out-of-hours work (39). An excessive workload would lead to decreased work performance, not conducive to effective health communication.

In macrosystem, the main influencing factors are national policies, regional characteristics and living environment. National policy requires family doctors to provide health management services for older residents (40), which is a fixed basis and requirement for village doctors to carry out health communication for older residents in rural areas. People live with family means different ways and contents of obtaining health information, living with a spouse is more beneficial for improving the health education of older residents than living with grandchildren (41). This may be due to the fact that children have limited time to communicate health information with them. The spouses of older people are generally concerned with the same content, making them more likely to care for each other. Older residents living in remote environments with fewer neighbors have a reduced frequency of communication with others, which is not conducive to receiving and sharing health information. Regional characteristics affect the elder’s acceptance of health communication. In this study, both the older and village doctors expressed that the residents in the area have a positive attitude toward health as a whole, it is conducive to family doctors to carry out health communication activities. Otherwise, it will have adverse effects.

### 4.1 Limitations

This study only explored the interpersonal health communication behavior of older people in rural areas of Ningxia Hui Autonomous Region, China. Because medical levels and health communication methods vary across different rural regions, further research is needed to select research subjects and access their applicability in other areas.

### 4.2 Implications for future policy

Based on the ecology of interpersonal health communication for rural older, corresponding measures can be developed at different levels. For instance, in microsystem and mesosystems, communication among older people individuals, family doctors, and their peers can be promoted. In exosystems, village health facilities can be improved to enhance the rural medical environment. In the macrosystem, efforts can be made to promote the integration of aging health service policies and communication channels, creating a synergistic policy effect.

### 4.3 Relevance for broader international audience of the findings

With the aging population, the demand for health management among the older people is increasing. This research, focusing on rural areas in China, aims to enhance international awareness among researchers and policymakers regarding the health knowledge acquisition of rural older people—a vulnerable group—and provide references for the subsequent implementation of health knowledge dissemination and management.

## Data Availability

The raw data supporting the conclusions of this article will be made available by the authors, without undue reservation.
